# A Hybrid Cooling Model Based on the Use of Newly Designed Fluted Conformal Cooling Channels and Fastcool Inserts for Green Molds

**DOI:** 10.3390/polym13183115

**Published:** 2021-09-15

**Authors:** Abelardo Torres-Alba, Jorge Manuel Mercado-Colmenero, Juan De Dios Caballero-Garcia, Cristina Martin-Doñate

**Affiliations:** Department of Engineering Graphics Design and Projects, University of Jaen, 23071 Jaen, Spain; ata00001@red.ujaen.es (A.T.-A.); jmercado@ujaen.es (J.M.M.-C.); jdcg0004@red.ujaen.es (J.D.D.C.-G.)

**Keywords:** injection molding, conformal cooling, industrial design, sustainability, green channels, numerical simulation, temperature maps

## Abstract

The paper presents a hybrid cooling model based on the use of newly designed fluted conformal cooling channels in combination with inserts manufactured with Fastcool material. The hybrid cooling design was applied to an industrial part with complex geometry, high rates of thickness, and deep internal concavities. The geometry of the industrial part, besides the ejection system requirements of the mold, makes it impossible to cool it adequately using traditional or conformal standard methods. The addition of helical flutes in the circular conformal cooling channel surfaces generates a high number of vortexes and turbulences in the coolant flow, fostering the thermal exchange between the flow and the plastic part. The use of a Fastcool insert allows an optimal transfer of the heat flow in the slender core of the plastic part. An additional conformal cooling channel layout was required, not for the cooling of the plastic part, but for cooling the Fastcool insert, improving the thermal exchange between the Fastcool insert and the coolant flow. In this way, it is possible to maintain a constant heat exchange throughout the manufacturing cycle of the plastic part. A transient numerical analysis validated the improvements of the hybrid design presented, obtaining reductions in cycle time for the analyzed part by 27.442% in comparison with traditional cooling systems. The design of the 1 mm helical fluted conformal cooling channels and the use of the Fastcool insert cooled by a conformal cooling channel improves by 4334.9% the thermal exchange between the cooling elements and the plastic part. Additionally, it improves by 51.666% the uniformity and the gradient of the temperature map in comparison with the traditional cooling solution. The results obtained in this paper are in line with the sustainability criteria of green molds, centered on reducing the cycle time and improving the quality of the complex molded parts.

## 1. Introduction

The plastic injection molding process is today one of the most widespread manufacturing processes due in large part to its efficiency and its ability to manufacture parts with a complex geometry at a competitive production cost [1]. The production process of parts by molding is a cyclical process that consists of four well-differentiated phases: the injection of the melt flow into the cavity, the packing of the plastic material until the gate freezes, the cooling of the plastic part until it solidifies, and finally, the ejection of the mold part [2]. Among these four phases, the cooling phase is the most influential in the production cost of the part as well as in the geometric and dimensional quality. The cycle time in the production of a mold is a measure of its productive efficiency [3]. A small decrease in the industrial cycle time leads to a large decrease in the energy expenditure of the injection molding process, making it more efficient and sustainable. However, achieving decreases in cycle time while maintaining the quality and requirements specified by the client is a highly complex process that involves studying in detail the heat-exchange process between surface layers, internal areas of the piece, and the fluid that circulates through the cooling channels.

The factors with the greatest influence on the heat exchange between molten plastic and coolant are the geometry, the layout, and the dimensions of the cooling channels. Injection molding cooling channels are traditionally manufactured using subtractive technologies such as CNC. The use of these technologies requires the use of dimensional technical specifications regarding the geometric design of the layout of the channels. In compliance with these specifications, it is necessary to guarantee the structural integrity of the mold against the high pressures and stresses to which it is subjected in its productive life.

Subtractive manufacturing technologies present high levels of material waste; therefore, unfortunately, they are not in line with current sustainability requirements [4,5]. The SLM additive manufacturing process allows for the manufacture of conformal channels adapted to the free shape of the geometric surface of the plastic part. In this way, it is possible to eliminate or reduce the existence of hotspots caused by differences in thickness, accumulations of material, or deep areas that are difficult to cool. The lack of uniformity in the cooling and, therefore, on the surface and internal temperatures of the piece can cause visible problems during its ejection or at a later time such as residual stresses, differential shrinkage, warpages, etc. In light of this, several authors have proposed different solutions regarding the geometry of the conformal channels with the aim of decreasing the cycle time, seeking energy savings in the process, and an improvement in the line of sustainability [6,7,8,9,10].

The layout of the cooling channels, topology, and sizing has a great influence on the variables of the heat-exchange process, such as pressure drop, cooling efficiency, coolant flow speed, etc. In the case of conformal cooling channels, their design allows them to be perfectly adapted to the requirements established by the geometry of the plastic part.

The spiral geometry is one of the most used topologies in the design of the layout in conformal cooling channels [11]. Linear zigzag geometry [12,13] is used in cases where spiral channels are difficult to implement. It should be noted that spiral geometry, in comparison to zigzag topology, presents sharp turns—increasing pressure drops, slowing the flow rate, and thus weakening the cooling efficiency. The application of the spiral-shaped and zigzag conformal channels decreases as the difficulty of the geometric surface of the plastic part to be manufactured increases. In these cases, mesh-topological conformal channels [14] and vascularized conformal systems [15] inspired by the design of blood vessels with complex topology and non-uniform diameter can be applied for complex pieces. Unfortunately, the design of a lattice topology conformal cooling system requires a detailed study regarding flow distribution and pressure drops since the usual design rules are useful for channel geometries with uniform diameters and shapes. In line with the admissible pressure drop in the layout, there is a minimum channel diameter below which the channel cannot be divided into sub-branches [16]. In addition, from a functional point of view, in the event of obstruction in the channels due to foreign bodies, it would be difficult to remove it due to the communication of the channels with each other.

When comparing the conformal cooling channels with respect to the geometry of their cross-section, it has been observed that the surface area of the conformal channels is an important parameter in the reduction of the cooling time and the improvement of the quality of the part [17]. Along these lines, although the circular cross-section is the most common in the design of conformal channels, research has been carried out to develop conformal channels with non-circular cross-sections such as square, rectangular, rhomboid, elliptical, water drop, etc., [18,19]. Several authors have made use of a square section for the cooling channels by making cooling slots in the mold [20,21]. The implementation of this solution is viable through traditional methods; however, it is difficult to produce with additive manufacturing since it requires supports that prevent the collapse of the material in the upper zone of the channel. In order to avoid deviations with the circular surface of the canal or even collapse in the upper zone, Kamat et al. [22] modified the circular section to a triangular self-supporting teardrop profile. The semicircular-shaped cooling channels consist of two parts, a semicircular part and another straight part parallel to the contour of the cavity. Unfortunately, although the semicircular conformal channel allows better tracking of the surface of the piece compared to the circular conformal channel and an improvement of heat dissipation [23,24], the sharp corner at the junction of the semicircular part and the straight part can cause stress concentration and crack propagation. Xi et al. [25], Wang et al. [26], and Jiang et al. [27] designed grooves on the inner surface of square channels in general cooling applications outside of injection molding. The ribs can enlarge the contact between the coolant and the channel surface. Along these lines, Freitas et al. [28] proposed a finned design on the circular or square channels to further expand the surface of the conformal channels; however, manufacturing the complex fin shape poses difficulties in additive manufacturing.

The steel used in the manufacture of molds and dies has disadvantages due to its low thermal conductivity regarding heat-transfer efficiency. Although increasing the coolant flow rate can increase the cooling efficiency, this option may be limited by the design of the mold, also leading to higher pumping costs [29]. Core cooling in molds can be performed with straight or perforated baffles in the mold core inserts. This results in a hollow, and consequently structurally weaker, core.

Another way to cool slender cores is to use inserts made of materials with high thermal conductivity, including copper, beryllium-copper, or high-strength sintered copper tungsten materials [30]. The high thermal conductivity of these materials increases the production speed while maintaining the strength to corrosion and oxidation, which is key for the lifetime of the mold [31]. Fastcool 50 is a hardened and tempered hot-work tool steel that has been recently developed with the aim of providing a tool steel with high thermal conductivity and high wear resistance at a reduced cost. Fastcool 50 material can go up to 54 Hrc while its thermal conductivity can reach almost twice that of conventional hot-work tool steels with the same level of hardness. It is especially indicated in applications subject to a high level of wear and in configurations in which the tool requires high thermal conductivity at high hardness [32]. Additionally, Fastcool 50 material allows for very high polish levels (mirror and higher), which are required in certain plastic injection molding applications. This tool steel allows one to considerably increase the productivity of the molding process as well as production problems related to the generation of hot/cold spots and poor distribution of the surface temperature.

Cycle time is currently the most important parameter in measuring the efficiency of the injection molding process [33,34,35]. The molding process is characterized by its high productivity in such a way that any reduction in cycle time brings great economic benefits to the company. The reduction of the cycle time depends to a great extent on the adaptability of the cooling channels to the complexity of the part. For a system of conformal channels located in problematic areas of the part, it is possible to reduce the cycle time by up to 30% compared to the use of conventional channels [36,37].

For a complete conformal channel system for the mold, the cycle time reduction can be more than 50% for parts with complex shapes and structures [38] or even 70% for some specific cases [39]. Several authors have investigated the influence of the use of conformal channels to reduce the cycle time. Shaifullah et al. [40] managed to reduce the cycle time by 35% with shaped cooling channels compared to straight cooling channels. In [41], they decreased the cycle time by 20% with a square section compared to conventional straight cooling channels. Xu et al. [42] applied a model based on unit cells for the dimensioning of conformal cooling, the results obtained by Xu indicated a 15% reduction of the cycle time. Colmenero et al. [43] performed a method to maximize the efficiency of shaped cooling channels manufactured by additive manufacturing. Research results showed that cooling and cycle time can be reduced by more than 50%. Additive manufacturing can create three-dimensional lattices and porous structures with specific mechanical and thermal properties. Mercado et al. developed a method based on the use of lattice to reduce the cycle time and improve the uniformity of the cooled part [44]. Using the design of porous structures, Brooks et al. [45] presented a study based on the use of shaped cooling layers designed with unsupported unit cells. These results showed a 26% decrease in cooling time compared to traditional cooling systems. Unfortunately, there are still major drawbacks in using uniform porous structures to achieve shaped cooling because the pressure drop in the porous structure is usually much greater than that of shaped cooling channels.

The design of a sustainable injection mold involves planning and optimizing its design by working on several lines simultaneously. First, the mold must be designed with the aim of minimizing the cycle time while maintaining uniformity in the surface temperatures of the part. In this way, it is possible to reduce the energy cost of production and rejections due to lack of quality. On the other hand, the mold must be designed and manufactured using technologies that reduce production wastes. Finally, the plastic part must use recycled plastic materials capable of meeting the requirements and specifications of the designed part.

In light of the problems posed in the state of the art, this paper presents a hybrid cooling model formed by a new design of fluted conformal channels in combination with the use of inserts designed with Fastcool material. The hybrid cooling design presented in the paper is shown as an alternative to traditional cooling design solutions with the aim of achieving high energy savings while maintaining a high level of quality in the manufacture of the part in line with the requirements of industrial sustainability. The research presented analyzes the thermal influence of the geometry of the conformal layout section for cases of complex geometry where the real space limitations given by the design of the manufacturing tooling and the topology of the part complicate the layout of the channels and the compliance with industry standards.

The hybrid cooling design developed in the paper was applied to an industrial piece with complex geometry with high rates of variation in its thickness and deep concavities in its internal area. Additionally, in the part presented, the design of the ejection system of the mold complicating the placement of the cooling channels, making it impossible to cool the part adequately by traditional methods. The results obtained in the research indicate that the introduction of the design with helical flutes in the conformal cooling channels improves the cycle time with respect to traditional cooling, above the geometries in circular, spiral, and water drop. Likewise, the transient numerical analysis carried out in this paper validates the combined use of fluted conformal cooling channels together with the use of Fastcool inserts for the areas with greater thickness, improving the temperature profile. The results obtained by the research are in line with the sustainability criteria in the design of green molds, capable of reducing the cycle time and manufacturing quality pieces with recycled plastic materials capable of meeting the high levels of quality and requirements established by the industrial customer.

## 2. Materials and Methods

### 2.1. Geometrical Design and Analysis for the Plastic Parts Manufactured through the Injection Molding Process

In this section, the geometrical, functional, and manufacturing features associated with the plastic part under study are described. Technical details regarding the selection of the plastic material for the injection molding manufacturing process, boundary conditions, and geometrical features are also specified. The piece under study is characterized by presenting a highly complex geometry, with a great influence on the performance of the molding manufacturing process. The piece presented in the paper corresponds to a real plastic piece of complex geometry manufactured industrially, belonging to a consumer product with a high annual production ratio. In industrial parts manufactured by the injection molding process, the parameters of cycle time and uniformity in cooling are indicators of the efficiency of the production process and the sustainability of the process. The injection molding process is a cyclical process; therefore, any decrease in cycle time implies high economic savings for the company, as well as an improvement in sustainability and energy savings.

**Figure 1:**
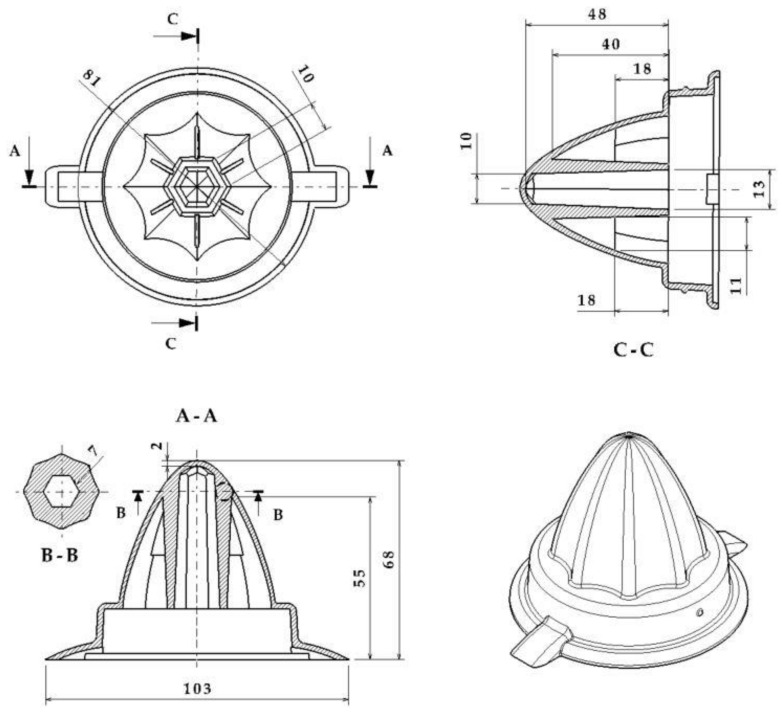
Description of the geometry of the plastic part. A-A section defined by A-A cutting plane, B-B section defined by B-B cutting plane and C-C section defined by C-C cutting plane.

Figure 1 shows the geometry of the industrial part. The dimensions of the bounding box of the part are 103 × 81 × 68 mm^3^. The piece has an interior area, characterized by the inclusion of a small hexagonal section blind hole. The blind hole crosses the part almost entirely; therefore, a reinforcement area is required in the upper area to avoid possible warpages and fractures in the part. Unfortunately, the reinforcing geometry creates a largely localized area over the thickness in the part due to material accumulation in that problem region. The maximum thickness in the reinforcement zone is 7 mm, while the rest of the piece has a general thickness of 2 mm (see Figure 1). Thus, the thickness ratio obtained for this case study is 3.5:1. Manufacturing a part by molding with this thickness gradient is a challenge since, as the thickness of the wall increases, so do the stresses and displacements associated with the effects of shrinkage. Additionally, the material accumulation zone has a greater heat accumulation due to a slower cooling process. This variation in the temperature of the part results in a prolonged cooling time and an uneven shrinkage, causing the part to warp heterogeneously. This accumulation of heat in the part also affects the productivity of the manufacturing process, as it causes a considerable increase in the total manufacturing cycle time. In addition, and as indicated in Figure 1, the geometry of the part has another added complication, which is the difficulty of access of the cooling to the problematic area. The reduced dimensions of the central hole of the piece do not allow the inclusion of traditional cooling channels while maintaining the structural integrity of the mold.

**Figure 2:**
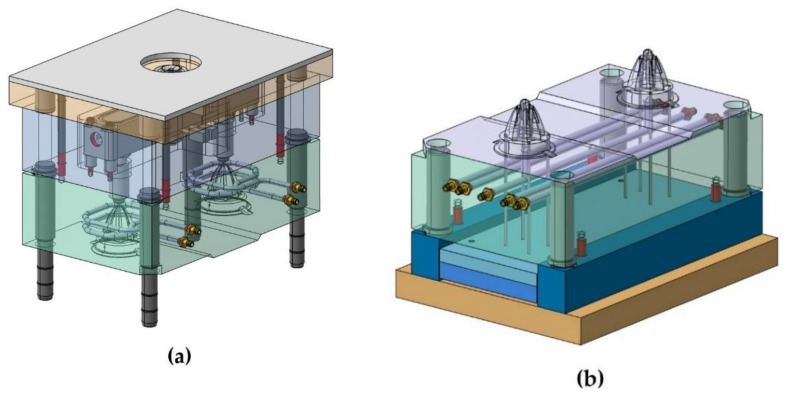
Mold of the plastic part injection area (**a**) and ejection area (**b**).

**Figure 3:**
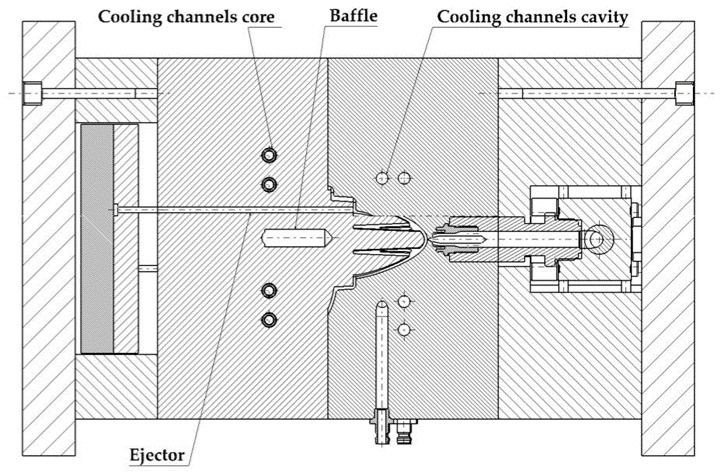
Mold cooling design using traditional straight channels and baffles.

The geometric design of the injection mold cooling system for the analyzed case study presents manufacturing difficulties for its main elements: perforated straight channels and baffles. The design of a traditional mold requires that the channels meet dimensional requirements that guarantee the structural integrity of the mold. These geometric requirements are related to the distance between channels, the distance to the ejectors, and the distance to the surface of the mold. The drills in charge of housing baffle-type cooling devices in the core must, in turn, meet a set of dimensional requirements related to the distance between channels, the surface of the part to be molded, and the ejectors of the system. Figure 2 shows the current design of the two-cavity mold following traditional manufacturing methods to manufacture the part under study. The mold cavity plate is cooled by a set of 8 mm diameter drilled straight holes which are located unevenly with respect to the surface of the mold, due to the requirements of traditional manufacturing using computerized numerical control (CNC) (see Figure 2). Likewise, the core plate is cooled using 6 mm diameter perforated straight channels and a 12 mm diameter and 40 mm deep baffle, located in the central part of the core of the plastic part (see Figure 2). The separation distance between channels themselves and the channels and the piece meets the criteria for sizing injection molds established by the industry, always being greater than the minimum safety distance that ensures the structural integrity of the mold. Figure 3 shows the assembly drawing of the mold, where the location of the cooling elements next to the ejectors is indicated. Figure 2 and Figure 3 show how the need to include four ejectors to extract the part from the mold greatly reduces the space available for cooling.

### 2.2. Green Conformal Cooling Channels Design

The cooling of concave cores in injection molds poses great difficulties in complex parts due, in large part, to the number of geometric and technological requirements caused by the manufacturing process. These requirements are sometimes difficult or impossible to solve using traditional cooling elements or systems.

Conformal cooling channels solve these problems, taking advantage of the free space between elements of the mold, reducing the distance to the surface of the plastic part in areas of difficult access and proposing new geometric designs and sections in the cooling channels.

Likewise, this manuscript analyzes in detail the influence that the geometry of the cooling channel section has on the heat-exchange process in industrial parts with highly complex geometric requirements. This manuscript presents a new conformal channel design, based on the introduction of helical flutes along the circular surface of conformal-type cooling channels. The objective of the flutes is to increase the turbulence of the coolant flow inside the cooling channel, thus improving the heat exchange between the plastic part and the coolant flow.

To evaluate the thermal performance of the fluted sections presented in this manuscript, three additional configurations of conformal channels were designed based on the use of circular, elliptical, and water drop geometries adapted to a spiral axis geometric sweep topology with which the geometric CAD modeling of the conformal cooling channels was carried out.

The dimensioning of the sections, the separation of the channels to the study piece, and the distance between channels were carried out in accordance with the requirements established in [46] and according to the topological requirements of the plastic piece. Although the geometric contour of the sections is variable, the proposed sections were designed in such a way that their hydraulic diameter D_h_ (see Equation (1) remains constant, maintaining the same number of turns in all the proposed designs. The hydraulic diameter D_h_ was calculated according to Equation [1], where A_s_ [m^2^] is the area of the cooling channel section and P [m] the perimeter of the channel section.

(1) $$D_{h}=4\cdot \frac{A_{s}}{P}$$

In this way, the thermal performance between the different geometric designs proposed is compared. The sections presented in this manuscript meet all the necessary requirements for their manufacture using additive technology. The dimensions and angles in the geometric contours of the channels presented meet the sustainability criteria regarding the design of green channels, avoiding the use of supports inside, as well as the elastic and structural collapse of the metal material of the injection mold. Table 1 indicates the values of the set of geometric and dimensional specifications used in the design of the layout of the different cooling channels applied in the cavity area of the mold.

**Figure 4:**
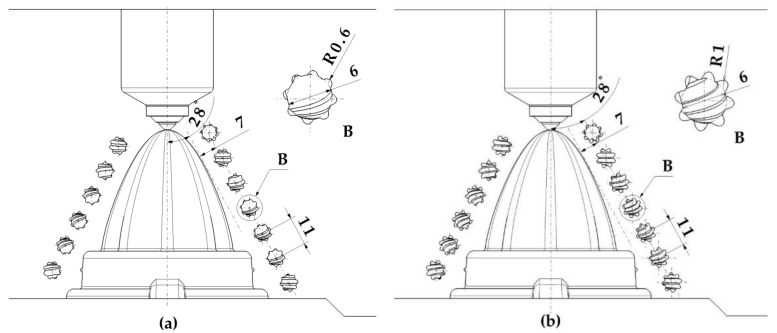
Design of the fluted conformal cooling channels (**a**) 0.6 mm and (**b**) 1 mm.

**Figure 5:**
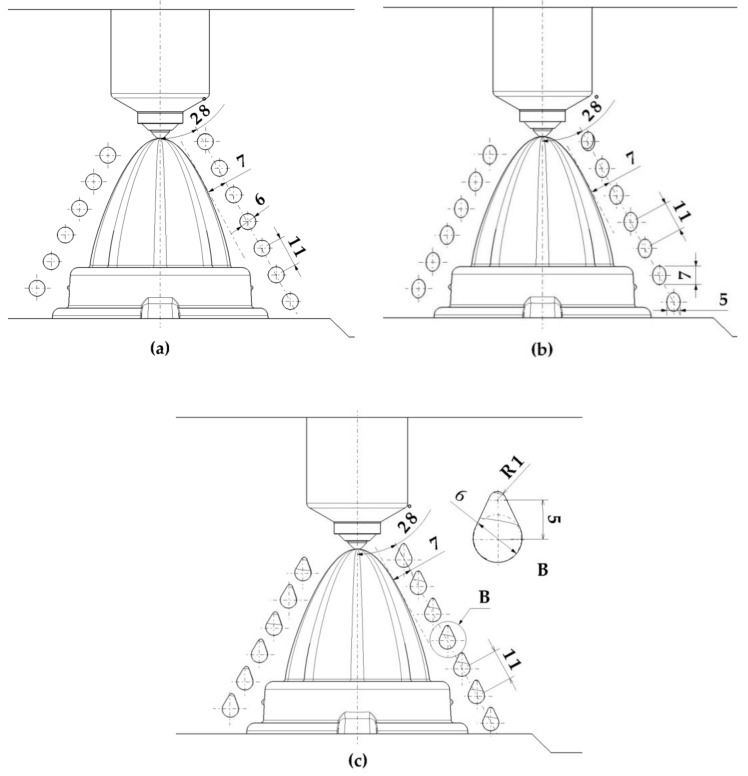
Conformal cooling channel design (**a**) circular, (**b**) elliptical, and (**c**) water drop.

Figure 4 presents the design of the fluted conformal cooling channels located in the cavity plate of the mold cavity. Figure 5 shows the design of the conformal channels with circular, elliptical, and water drop sections, respectively.

Although conformal cooling is suitable for cooling complex areas, sometimes the geometry of the plastic part has space limitations that make it difficult to place conformal channels. This problem usually occurs when cooling thin areas and geometric regions with deep cores. The plastic piece under study is framed within this last group, presenting great geometric limitations characterized by internal areas of difficult access together with a central hole of great depth and width 10 mm.

**Figure 6:**
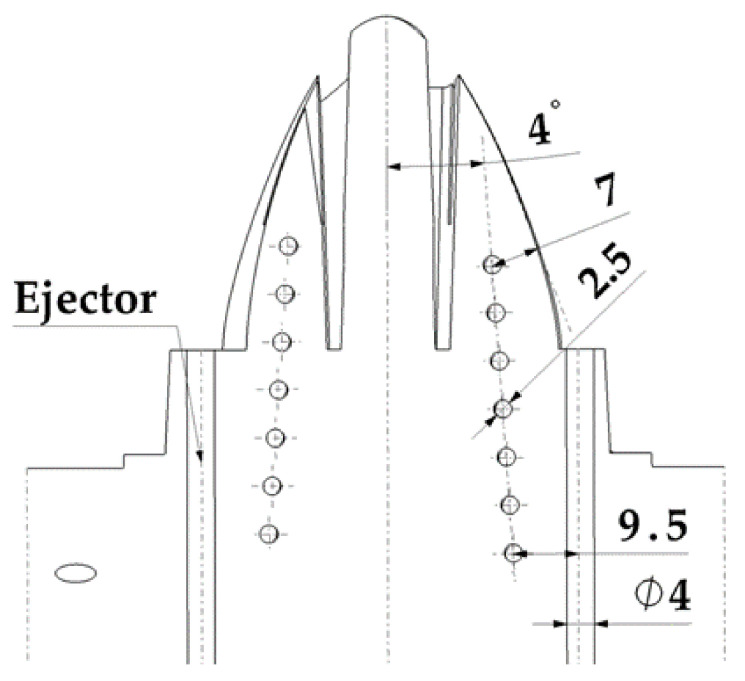
Design of conformal cooling channels in the core area.

This geometry prevents the use of conformal green channels in the upper area with a higher thickness ratio. Since, firstly, there is not enough space in the core to access the channels, and secondly, the diameter of the channels would be too small to provide functionality in cooling. Figure 6 shows the layout corresponding to the conformal channels designed in the core area. Unfortunately, as seen in Figure 6 for geometric and dimensional reasons, it is not possible to cool the internal area of the core using conformal type channels.

**Figure 7:**
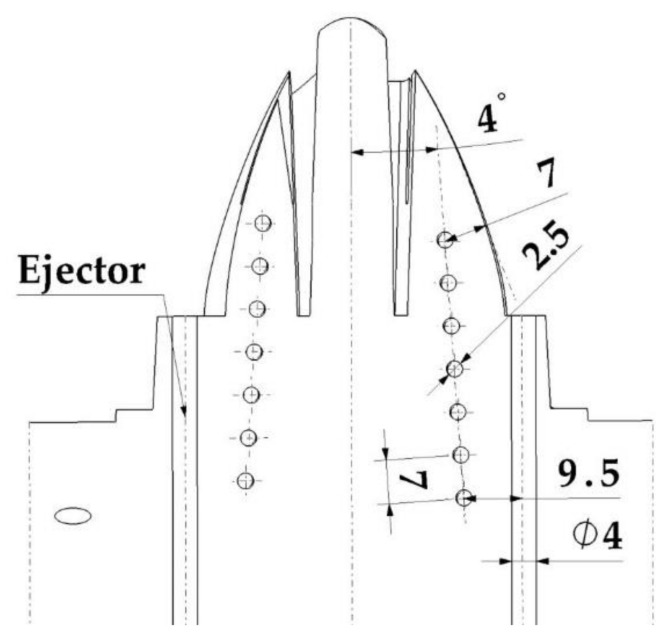
Hybrid Fastcool-conformal cooling system for core cooling.

**Figure 8:**
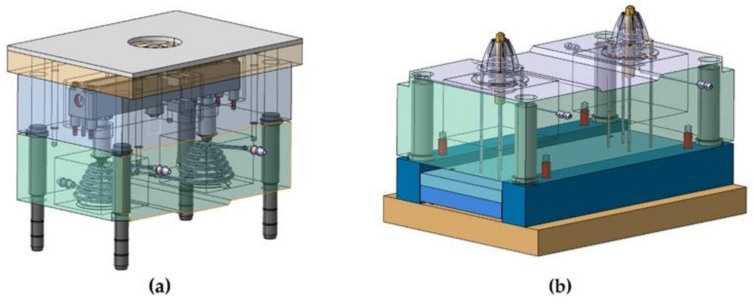
Final design of the mold including the hybrid cooling proposed in this paper. Mold of the plastic part injection area (**a**) and ejection area (**b**).

To solve the problem posed, this manuscript presents a hybrid cooling design, based on the use of Fastcool inserts in combination with conformal-type cooling systems. Fastcool inserts are characterized by their high thermal transmission coefficient. This allows the heat exchange in high-temperature regions in the plastic part to be greatly accelerated and improved. However, the area of the mold in contact with the insert accumulates a large amount of heat flow that the insert is not able to dissipate, generating local points with a great accumulation of heat and high temperatures. The use of conformal cooling channels, in these cases, can be very useful, not so much for cooling the plastic part directly but for cooling the Fastcool insert itself, above all, in those plastic pieces in which the use of conformal channels is not enough. In this way, it is possible to minimize the cooling time of the part making the molding process sustainable, while maintaining the surface quality of the molded part. Table 2 shows the geometric data of the conformal channel used for core cooling and the Fastcool insert. Figure 7 presents the application of the hybrid Fastcool-conformal cooling system presented in this manuscript. Figure 8 shows the final design of the mold.

### 2.3. Materials

The piece under study was designed to be manufactured with recycled PP 108MF10 thermoplastic material from the SABIC company. This plastic material is obtained from a chemical recycling process. Therefore, the mechanical, thermal, and chemical properties of the original plastic are maintained without compromising the sustainability of the injection process. The magnitudes of the main properties of the material PP 108MF10 are indicated in Table 3. Table 4 and Table 5 show the features of the metal material of Fastcool and the injection mold metal material. The material properties shown in these tables are provided by their manufacturers. Furthermore, the presented cooling system design methodology is universal; therefore, it can be applied to any thermoplastic material used in the manufacture of plastic parts through the injection mold manufacturing process.

## 3. Implementation and Results

The geometric designs of the different cooling channels proposed in this manuscript were developed and analyzed numerically in the CAD design software Catia (V5-6R2020 version, Dassault Systèmes, Vélizy-Villacoublay, Francia) [47] and numerical analysis Moldex3D (R17 version, CoreTech System Co., Ltd, Zhubei, Taiwán) [48] with an MSI notebook (Micro-Star International, Co., Ldt, Taipei, Taiwán) with an Intel (R) Core (TM) i-77700HQ CPU @ 2.80 GHz (Intel Corporation, Santa Clara, CA, US). The geometry of the cooling channels, especially the design of the conformal cooling channels, was parameterized and adapted to the geometric features of the case study and the technical and technological requirements imposed by the 3D additive manufacturing process by laser sintering (SLS). This parameterization, together with the modeling of the layout of the conformal cooling channels, was automated by generating an application in the programming environment of the CAD software Catia (V5-6R2020 version, Dassault Systèmes, Vélizy-Villacoublay, Francia) [47].

### Modeling of Numerical Simulations for the Thermal Analysis of the Green Conformal Cooling System

**Figure 9:**
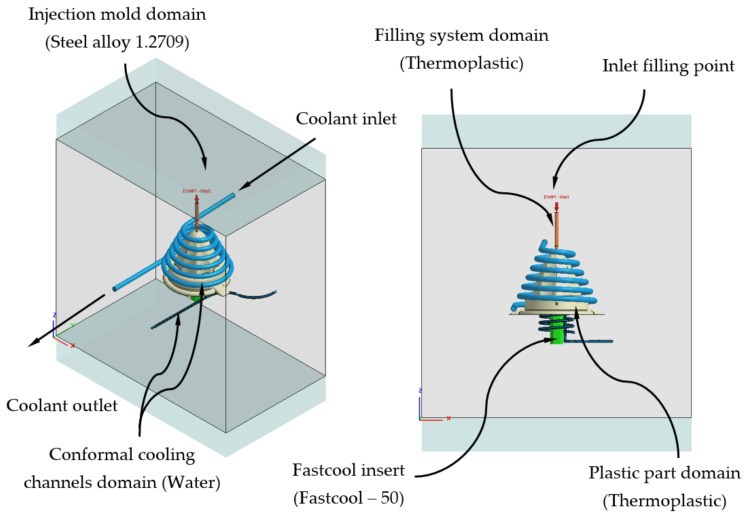
Domains and boundary conditions definition for the numerical simulations, conformal cooling solution with Fastcool insert.

In this section, we proceed to detail the pre-processing and modeling process of the different analyses and numerical simulations, from which the thermal performance of the designs of the conformal cooling channels and the rheological parameters associated with the process are evaluated for the manufacturing of the plastic part under study. Through this set of numerical analyses, the thermal and dynamic behavior of the coolant flow along the cooling channels is analyzed, as well as the thermal exchange produced between them, the plastic part, the Fastcool insert (see Figure 9), and the injection mold during the cooling phase of the plastic part. In this way, it can be validated if the thermal performance of the results obtained meets the technological requirements demanded by the manufacturing process using plastic injection molds. The set of numerical, thermal, and rheological analyses were carried out using the commercial simulation software CAE Moldex3D (R17 version, CoreTech System Co., Ltd, Zhubei, Taiwán) [48]. First, to describe the preprocessing modeling of this set of numerical analyses, five main 3D computation domains must be established (see Figure 9), along with the selection of the material associated with each of these. Likewise, these computational domains are established: plastic part (PP108MF10), feeding system (PP108MF10), cooling system (Water), injection mold (Steel alloy 1.2709), and Fastcool insert (Fastcool-50 at 44 HRC). Table 3, Table 4, and Table 5 show the main physical, rheological, and thermal properties of the materials used for each computational domain.

Moreover, together with the definition of the 3D computational domains that makes up the modeling of the numerical analyses, a series of main premises that complete the pre-processing phase are determined:

- Since the complete cooling process of the plastic part is analyzed over time, the type of numerical analysis used is “Cooling transient”.
- The total cooling time established for each numerical analysis is 90 s, with a time step between each time step of 10 s. For each defined time step, the numerical analysis software stores the solution obtained. Therefore, in each numerical simulation carried out, the time until reaching the ejection temperature of the plastic part and the evolution of the temperature map throughout the cooling phase can be precisely determined.
- The analysis of the behavior and evolution of the physical, dynamic and thermal properties of the coolant flow along the channels of the cooling system was modeled according to the “Run 3D cooling channels” configuration.
- The methodology used to configure the solver, in the resolution of each numerical analysis carried out, is of the type maximum variation of mold temperature, whose magnitudes established for the parameter temperature difference and maximum cycle number are 1 and 10 °C, respectively.
- The turbulence model used for the development of the numerical analyses is established using the roughness parameter. This parameter defines the interface surface between the coolant flow and the surface of the cooling channels. The magnitude defined for this technological parameter is equal to 0.02 mm

**Figure 10:**
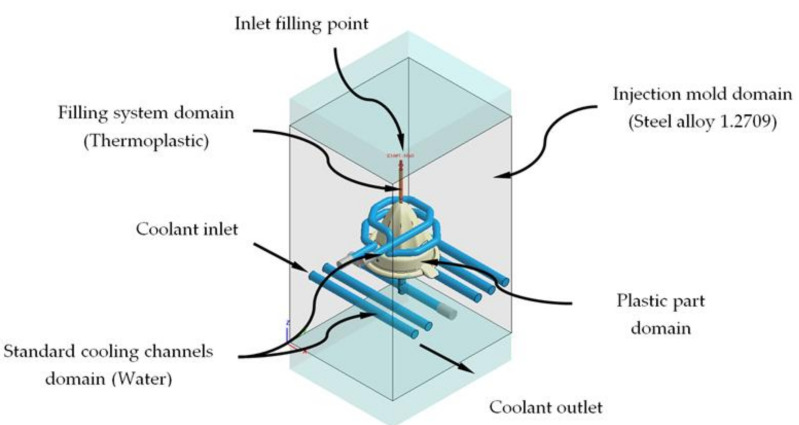
Domains and boundary conditions definition for the numerical simulations, traditional cooling solution.

Next, we proceed to the discretization of the main geometry of the cooling channels, plastic part, and injection mold (see Figure 9 and Figure 10) in finite volumes. The commercial software Moldex 3D R17 [48] has a Moldex Designer meshing module, from which the main parameters of the mesh are configured and defined for each numerical analysis. Table 6 shows the magnitude of the parameters defined for the meshing process, as well as its configuration.

**Figure 11:**
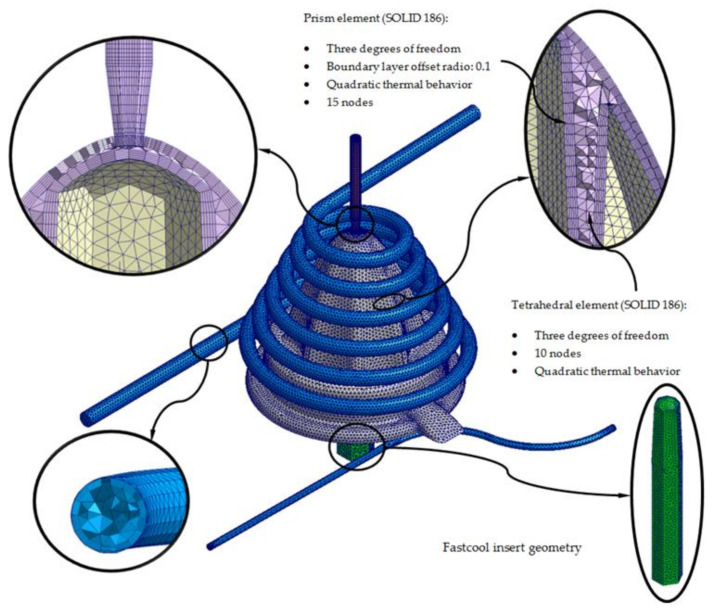
Mesh details for the conformal cooling solution.

**Figure 12:**
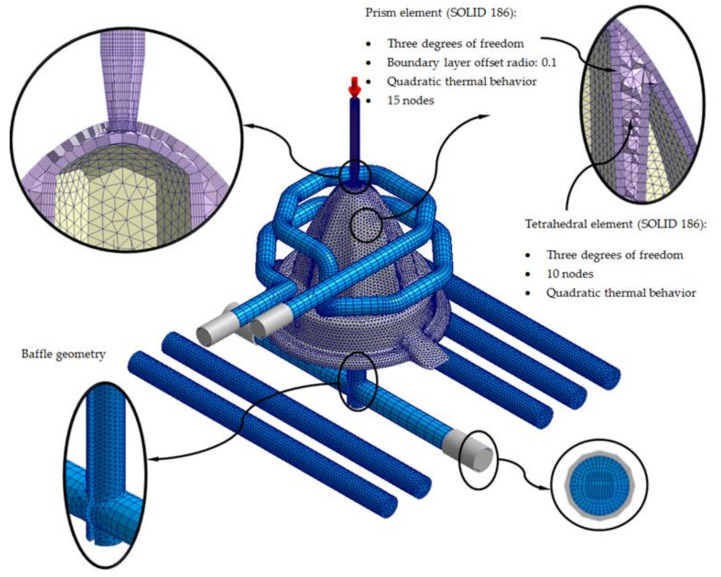
Mesh details for the traditional cooling solution.

Figure 11 and Figure 12 show in detail the typology of elements used to discretize, in finite volumes, the 3D computation domains presented in Figure 9 and Figure 10. The said elements are of the second order tetrahedron type (SOLID 186); they present 10 nodes of control of which, four are located at the vertices of the tetrahedron, and six are located at the midpoints of the edges. Being a second-order element, each node has 3 degrees of freedom, with translation in the main coordinate axes. The use of this type of element allows the resulting temperature field to be modeled with greater precision.

Furthermore, in order to improve the precision of the numerical simulations, five layers of second-order prismatic elements (SOLID 186) of the “Boundary Layer” type are defined for the interface surfaces, along with the injection mold geometry “Mesh” (see Figure 11 and Figure 12). These elements have 15 nodes; six are located at the vertices of the tetrahedron, and nine are located at the midpoints of the edges. Being a second-order element, each node has 3 degrees of freedom, with translation in the main coordinate axes. The magnitude of these elements is set by the offset ratio parameter, which is defined as a percentage of the average size of the mesh element. Table 6 shows the mesh statistics for the standard and green conformal cooling systems.

For each numerical analysis performed, a set of boundary conditions related to the technological parameters that determine the molten plastic front and the coolant flow at the beginning of the plastic injection manufacturing process were established. For the cooling channels, an inlet and outlet surface of the coolant flow is established, as well as the magnitude of the technological parameters of inlet temperature and pressure of the coolant flow (see Table 7). On the other hand, for the cooling system the input surface of the molten plastic front is established, as well as the magnitude of the technological parameters of temperature, pressure and flow of the molten plastic front (see Table 7). It should be noted that, for the dimensioning of the coolant flow inlet pressure variable to the refrigeration channels, a quantity was established that ensures and allows the coolant flow front to develop in a turbulent regime. In other words, the Reynolds number of the coolant flow along the cooling channels is greater than 1.5 × 10^4^. This condition was kept constant for each of the numerical simulations carried out, maintaining in each of them a Reynolds number equal to 5.0 × 10^4^. Table 7 shows technological variables defined for the set-up of the filling and cooling stage for numerical simulations, the magnitude of the technological parameters presented are those recommended by the manufacturer of the plastic material.

It should be noted that, to perform the validation of the numerical simulations carried out, the technological parameters defined as an input during the preprocessing phase (see Table 7) were determined, validated, and contrasted by means of experimental tests. These tests were performed by the manufacturer and supplier of the thermoplastic material using the standard manufacturing technique of plastic injection on industrial molds.

After describing the modeling process of each of the numerical simulations carried out for the present case study, we proceeded to present the results obtained. From the evaluation, analysis, and validation of the case study, the geometry of the conformal cooling channel that optimizes the cooling phase of the plastic part and improves the thermal performance of the cooling system can be established. Likewise, the improvement in the efficiency and thermal performance obtained by using a Fastcool insert to cool the core area of the plastic part which presents cooling difficulties due to its high temperatures is verified. It should be noted that each of the configurations of conformal cooling systems analyzed and proposed was numerically simulated including the Fastcool type for the core area of the plastic part under study.

**Figure 13:**
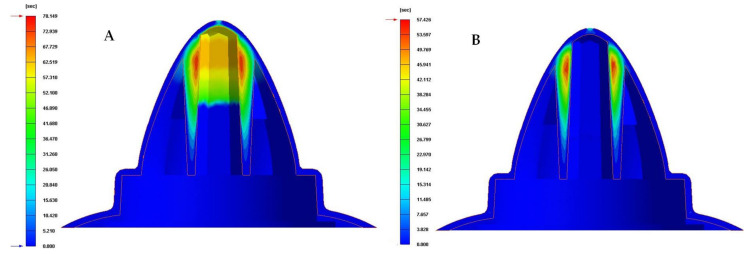
Time to reach ejection temperature (s). (**A**) Traditional cooling. (**B**) Circular conformal cooling channels and Fastcool insert.

**Figure 14:**
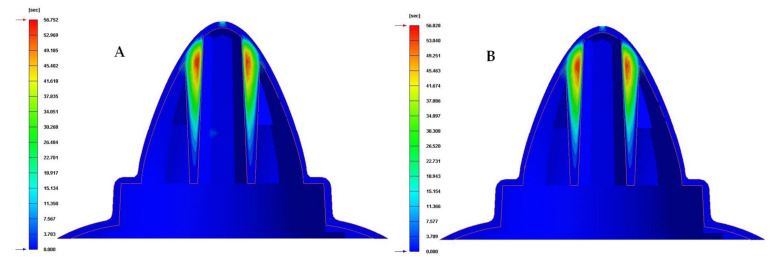
Time to reach ejection temperature (s). (**A**) Elliptical conformal cooling channels and Fastcool insert. (**B**) Water drop conformal cooling channels and Fastcool insert.

**Figure 15:**
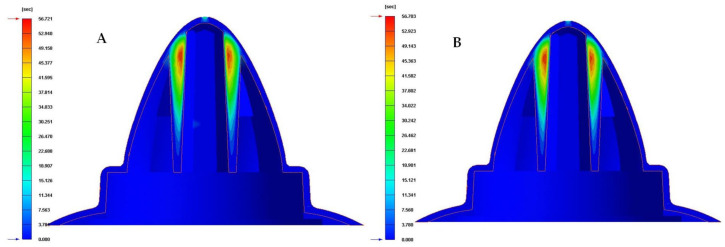
Time to reach ejection temperature (s). (**A**) Fluted 0.6 mm conformal cooling channels and Fastcool insert. (**B**) Fluted 1 mm conformal cooling channels and Fastcool insert.

In the first place, Table 8 and Figure 13, Figure 14 and Figure 15 show the results obtained for the parameter time until reaching the ejection temperature of the plastic part, for each of the cooling system configurations proposed.

As can be seen in Table 8 and Figure 13, Figure 14 and Figure 15, the configurations of the conformal cooling systems that incorporate the Fastcool insert improve the time until reaching the ejection temperature in the plastic part. In particular, the conformal cooling channel solution with a fluted section of 1 mm is the one that minimizes this parameter because the time to reach the ejection temperature is reduced by 21.446 s in comparison to the traditional solution defined by straight drilled channels. This represents an improvement in the manufacturing cycle time of the plastic part under study of 27.4%. In addition, the thermal performance of the conformal cooling channel with a fluted section of 1 mm improves on the rest of the proposed conformal cooling solutions. This result is due to the geometry of the flutes along the channels surface generating vortices and turbulence in the coolant flow, which help and favor it to develop and constantly maintain a turbulent regime.

**Figure 16:**
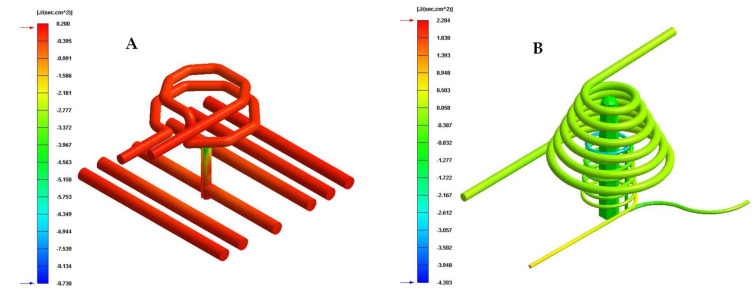
Heat flow (J/s·cm^2^). (**A**) Traditional cooling. (**B**) Circular conformal cooling channels and Fastcool insert.

**Figure 17:**
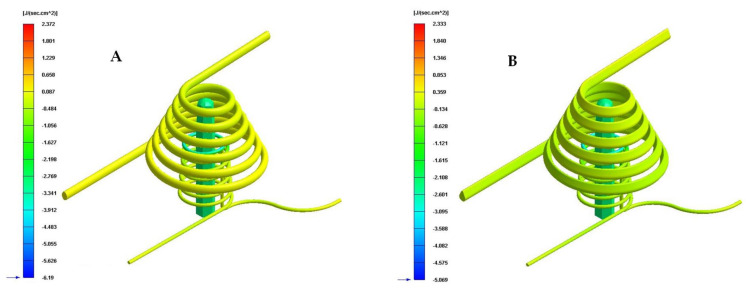
Heat flow (J/s·cm^2^). (**A**) Elliptical conformal cooling channels and Fastcool insert. (**B**) Water drop conformal cooling channels and Fastcool insert.

**Figure 18:**
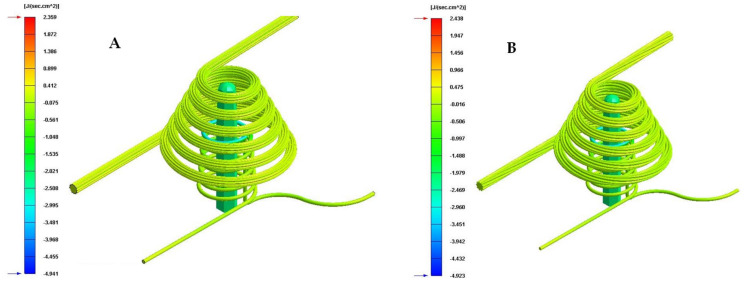
Heat flow (J/s·cm^2^). (**A**) Fluted 0.6 mm conformal cooling channels and Fastcool insert. (**B**) Fluted 1 mm conformal cooling channels and Fastcool insert.

Table 9 and Figure 16, Figure 17 and Figure 18 show the magnitude of the heat flow that is exchanged between the computational domain of the cooling channels and the rest of the domains defined for each of the numerical simulations performed.

As can be seen in Table 9 and Figure 16, Figure 17 and Figure 18, the design of some conformal-type cooling channels together with the implementation of a Fastcool insert, used for cooling the plastic part, represents a relevant improvement in the heat exchange that takes place between the coolant flow and the plastic part. On the one hand, for the cavity plate of the injection mold, the cooling channel geometry that exchanges the greatest amount of heat flow with the plastic part is the 1 mm fluted channel. The definition of flutes along the surface of the channel, with a dimension of 1 mm, favors the development of the coolant flow in a turbulent regime and, therefore, the thermal exchange of said fluid. On the other hand, for the core plate of the injection mold, the use of a Fastcool insert allows for the optimal evacuation and transfer of heat flow in the core of the plastic part. However, the Fastcool insert is not capable of maintaining, by itself, a constant heat exchange throughout the manufacturing cycle of the plastic part. For this reason, a conformal type cooling channel layout was defined, which allows establishing a thermal exchange between the Fastcool insert and the coolant flow. In this way, it is determined that the design of the 1 mm fluted type cooling channels together with the use of a Fastcool insert cooled by a conformal type channel improves by 4335% and 2.731 [J/s·cm^2^] the thermal exchange between the cooling elements and the plastic part, with respect to the configuration of the traditional cooling system.

**Figure 19:**
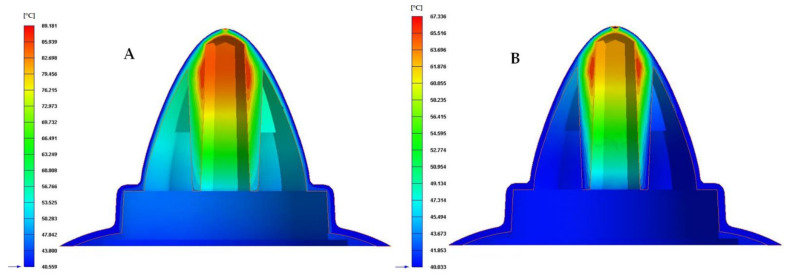
Cooling temperature (°C). (**A**) Traditional cooling. (**B**) Circular conformal cooling channels and Fastcool insert.

**Figure 20:**
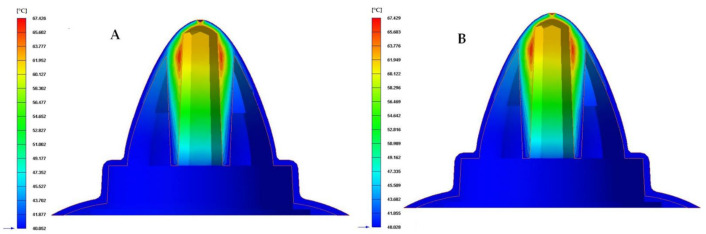
Cooling temperature (°C). (**A**) Elliptical conformal cooling channels and Fastcool insert. (**B**) Water drop conformal cooling channels and Fastcool insert.

**Figure 21:**
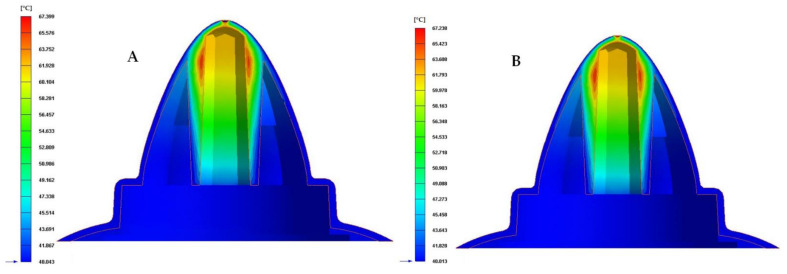
Cooling temperature (°C). (**A**) Fluted 0.6 mm conformal cooling channels and Fastcool insert. (**B**) Fluted 1 mm conformal cooling channels and Fastcool insert.

Finally, to complete the analysis of the results obtained in the different numerical simulations carried out, the temperature map after the cooling phase for each proposed cooling system is presented in Figure 19, Figure 20 and Figure 21. Table 10 shows the maximum temperature difference along the surface of the plastic part under study.

From the results obtained for the temperature maps, it was established that the design of a conformal type cooling system, accompanied by a Fastcool insert, improves the uniformity and gradient of the temperature map along the geometry of the plastic part object of study. Specifically, this improvement ranges from 49.4% to 51.7% and 22.093 to 23.109 °C, depending on the section of the defined conformal-type cooling channels. Being the 1 mm fluted type cooling channel, the one with the greatest uniformity of the temperature map and the lowest maximum temperature generated on the plastic piece was studied in this manuscript.

It should be noted that, the performance improvement parameter (see Table 8, Table 9, and Table 10) is computed as the percentage that represents the magnitude of the variables time reduction [s] (see Table 8), total improvement [J/s·cm^2^] (see Table 9), and total improvement [°C] (see Table 10), obtained for each cooling system design proposed in this manuscript, on the magnitude of the numerical solution obtained for the traditional cooling system for each of parameters analyzed: Time to reach ejection temperature [s] (see Table 8), Heat flow [J/s·cm^2^] (see Table 9), and Mold temperature difference [°C] (see Table 10).

## 4. Conclusions

The paper presents a hybrid cooling model based on the use of newly designed fluted conformal cooling channels in combination with inserts manufactured with Fastcool material. The hybrid cooling design was applied to an industrial part with complex geometry, high rates of thickness, and deep internal concavities. The geometry of the industrial part, besides the ejection system requirements of the mold, makes it impossible to cool it adequately using traditional or conformal standard methods. 

The addition of helical flutes in the conformal cooling channel surface generates a high number of vortexes and turbulences in the coolant flow, helping and fostering the thermal exchange between the flow and plastic part. The use of a Fastcool insert allows for the optimal evacuation and transfer of the heat flow in the slender core of the plastic part. An additional conformal cooling channel layout was required, not for cooling the plastic part, but for cooling the Fastcool insert improving the thermal exchange between the Fastcool insert and the coolant flow. In this way, it is possible to maintain a constant heat exchange throughout the manufacturing cycle of the plastic part. 

A transient numerical analysis carried out validates the improvements of the hybrid design, presenting reductions in cycle time by 27.442% and 21.446 s for the complex plastic part analyzed in comparison with the results obtained from traditional cooling systems. The design of the 1 mm fluted conformal cooling channels and the use of the Fastcool insert cooled by a conformal cooling channel improves, by 4334.9%, the thermal exchange between the cooling elements and the plastic part in comparison with traditional cooling systems.

From the results of the plastic part temperature map, it was established that the conformal cooling system accompanied by a Fastcool insert improves the uniformity and gradient of the temperature map in ranges from 49.394% to 51.666% and 22.093 to 23.109 °C in comparison to the traditional cooling solution. The design of a 1 mm fluted conformal cooling channel allows the greatest temperature map uniformity and the lowest maximum temperature on the plastic part studied in this manuscript.

The hybrid cooling design is shown as an alternative to traditional and standard conformal cooling systems for complex geometrical parts. In this way, it is possible to achieve a green mold with high energy savings and quality parts following the current requirements of sustainability in the plastic industry. Although there are other authors who have carried out experiments so far in the study of the application of conformal cooling circuits to improve cycle time, these authors have focused on simple geometry pieces and the use of standard conformal cooling circuits. These conformal designs are not suitable for the parts of complex geometry as presented by the authors in their research.

The results obtained by the research are in line with the sustainability criteria for green molds, centered on reducing the cycle time and improving the quality of the molded parts using recycled plastic materials, meeting the requirements established by the industrial customers.

## Figures and Tables

**Table 1:** Geometric and dimensional specifications used in the design of the layout of the different channels in the cavity plate of the mold.

Nomenclature	Units	Circular	Elliptical	Water Drop	Fluted Circular 1 mm	Fluted Circular 0.6 mm
A_s_	mm^2^	28.27	27.49	35.93	39.27	39.27
P	mm	18.84	18.98	22.77	30.25	27.76
D_h_	mm	6	5.79	6.31	5.19	5.66
s	mm	7	7	7	7	7
p	mm	11	11	11	11	11

where A_s_ [mm^2^] represents the area of the cooling channel section, P [mm] represents the perimeter of the cooling channel, D_h_ [mm] represents the hydraulic diameter of the cooling channel section, s [mm] represents the minimum distance between the center of the cooling channel section and the surface of the plastic part, and p [mm] represents the distance between centers of the cooling channel sections.

**Table 2:** Geometric parameters used in the design of conformal cooling channels.

Nomenclature	Units	Description	Circular
A_s_	mm^2^	Section area	4.9
P	mm	Perimeter	7.85
D_h_	mm	Hydraulic diameter	2.5
s	mm	Distance channel center—mold surface	7
p	mm	Pitch between channels	7

**Table 3:** Magnitude of the main properties of the material PP108MF10.

Nomenclature	Units	Description	PP 108MF10
ρ_p_	Kg/m^3^	Density	905
C_p_	J/Kg·°C	Specific heat	2704
δ_p_	W/m·°C	Thermal conductivity coefficient	0.1998
T_melt_	°C	Melt temperature (normal)	230
T_mold_	°C	Mold temperature (normal)	40
T_eject_	°C	Ejection temperature	100

**Table 4:** Mechanical, physical, and thermal properties of Fastcool-50 at 44 HRC.

Description	Units	Fastcool-50
Density	g/cm^3^	7.81
Yield strength 0,2%	MPa	1070
Mechanical resistance	MPa	1400
Elongation	%	17
Specific heat capacity	J/g·K	0.47
Thermal diffusivity	mm^2^/s	13.5
Thermal conductivity	(W/m·K)	50

**Table 5:** Mechanical, physical, and thermal properties of Steel alloy 1.2709.

Description	Units	Steel Alloy 1.2709
Density	g/cm^3^	8000
Specific heat capacity	J/g·K	450
Thermal conductivity coefficient	(W/m·K)	20

**Table 6:** Mesh statistics for the standard and conformal cooling meshes.

Description	Units	Value
Part mesh node count	-	107,872
Part mesh element count	-	236,119
Part mesh volume	cm^3^	33.89
Runner mesh node count	-	14,701
Runner mesh element count	-	13,440
Runner mesh volume	cm^3^	0.5
Plastic part precision (ε)—Mesh sizing	mm	1.5
Element type	-	Tetrahedral (10 nodes)
Element type—Boundary layers	-	Prism (15 nodes)
Offset ratio—Boundary layers	-	0.1

**Table 7:** Technological variables defined for the setup of the filling and cooling stage for numerical simulations.

Description	Units	Study Cases—PP 108MF10 (PP)
Filling time	s	1.19
Packing time	s	5.91
Cooling time	s	90
Melt temperature	°C	230
Mold temperature	°C	40
Ejection temperature	°C	100
Coolant temperature	°C	40
Maximum injection pressure	MPa	140
Packing pressure profile	MPa	85 (0.0–3.5 s) 40 (3.5–4.7 s) 10 (4.7–5.9 s)

**Table 8:** Time to reach ejection temperature for each proposed cooling system configurations.

Cooling System Configurations	Time to Each Ejection Temperature [s]	Time Reduction [s]	Performance Improvement [%]
Traditional	78.149	-	-
Circular	57.426	20.723	26.5
Water drop	56.826	21.323	27.3
Elliptical	56.752	21.397	27.4
Fluted 0.6 mm	56.721	21.428	27.4
Fluted 1 mm	56.703	21.446	27.4

**Table 9:** Heat flow for each proposed cooling system configurations.

Cooling System Configurations	Cavity Cooling [J/s·cm^2^]	Core Cooling [J/s·cm^2^]	Fastcool Insert [J/s·cm^2^]	Total [J/s·cm^2^]	Total Improvement [J/s·cm^2^]	Performance Improvement [%]
Traditional	0.004	0.059	-	0.063	-	-
Circular	0.046	0.078	2.284	2.408	2.471	3722
Raindrop	0.195	0.085	2.333	2.613	2.550	4048
Elliptical	0.268	0.017	2.372	2.657	2.594	4118
Fluted 0.6 mm	0.206	0.103	2.359	2.668	2.605	4135
Fluted 1 mm	0.238	0.118	2.438	2.794	2.731	4335

**Table 10:** Mold temperature difference [°C] for each proposed cooling system configurations.

Cooling System Configurations	Mold Temperature Difference [°C]	Total Improvement [°C]	Performance Improvement [%]
Traditional	44.728	-	-
Circular	22.635	22.093	49.4
Water drop	22.432	22.296	49.8
Elliptical	22.450	22.278	49.8
Fluted 0.6 mm	21.644	23.084	51.6
Fluted 1 mm	21.619	23.109	51.7

## Data Availability

All data included in this study are available upon request by contact with the corresponding author.
